# Consanguineous familial study revealed biallelic *FIGLA* mutation associated with premature ovarian insufficiency

**DOI:** 10.1186/s13048-018-0413-0

**Published:** 2018-06-18

**Authors:** Beili Chen, Lin Li, Jing Wang, Tengyan Li, Hong Pan, Beihong Liu, Yiran Zhou, Yunxia Cao, Binbin Wang

**Affiliations:** 1 Department of Obstetrics and Gynecology, Reproductive Medicine Center, The First Affiliated Hospital of Anhui Medical University, Meishan Road, Shushan, Hefei, 230022 China; 2 Central Laboratory, Beijing Obstetrics and Gynecology Hospital, Capital Medical University, Chaoyang, Beijing, 100026 China; 3 Department of Medical Genetics and Developmental Biology, School of Basic Medical Sciences, Capital Medical University, No. 10 Xitoutiao, Youanmenwai, Fengtai, Beijing, 100069 China; 4 Center for Genetics, National Research Institute for Family Planning, 12 Dahuisi Road, Haidian, Beijing, 100081 China; 5 Institute of Reproductive Genetics, Anhui Medical University, Meishan Road, Shushan, Hefei, 230032 China; 6 Anhui Provincial Engineering Technology Research Center for Biopreservation and Artificial Organs, Meishan Road, Shushan, Hefei, 230027 China; 7 Key Laboratory of Family planning and Reproductive Genetics, National Health and Family Planning Commission, Heb Research institute For Family Planning, Beijing, 050071 People’s Republic of China

**Keywords:** Premature ovarian insufficiency, FIGLA, Whole-exome sequencing

## Abstract

**Background:**

To dissect the genetic alteration in two sisters with premature ovarian insufficiency (POI) from a consanguineous family.

**Methods:**

Whole-exome sequencing technology was used in the POI proband, bioinformatics analysis was carried out to identify the potential genetic cause in this pedigree. Sanger sequencing analyses were performed to validate the segregation of the variant within the pedigree. In silico analysis was also used to predict the effect and pathogenicity of the variant.

**Results:**

Whole-exome sequencing analysis identified novel and rare homozygous mutation associated with POI, namely mutation in *FIGLA* (c.2 T > C, start codon shift). This homozygous mutation was also harbored by the proband’s sister with POI and was segregated within the consanguineous pedigree. The mutation in the start codon of the *FIGLA* gene alters the open reading frame, leading to a *FIGLA* knock-out like phenotype.

**Conclusions:**

Biallelic mutations in *FIGLA* may be the cause of POI. This study will aid researchers and clinicians in genetic counseling of POI and provides new insights into understanding the mode of genetic inheritance of *FIGLA* mutations in POI pathology.

## Background

Premature ovarian insufficiency (POI) is a severe disorder of ovarian dysfunction and is characterized as decreased ovarian reserve and increased follicle-stimulating hormone (FSH) level. The aetiology is complex, among which the genetic alteration is one of the causes of POI and includes X chromosomal abnormalities, balanced translocations, and fragile X mental retardation 1 (*FMR1*) premutations and single gene defects [1]. Although several single gene variants have been associated with POI in recent decades [1], only a few have been proven to cause POI, e.g., *FSHR*, *BMP15*, *NOBOX*, *MCM8*, *MCM9*, *STAG3*, *HFM1*, *MSH4*, and *MSH5* [2]. Recently, we and another groups suggested that some genes and mutations cause POI in an autosomal recessive mode of inheritance rather than previous suggested heterozygous manner [3–6]. Together, these findings have expanded our knowledge on POI and helped distinguish causative mutations and risk alleles for POI.

In recent years, whole-exome sequencing (WES) technology has become a powerful tool for elucidating the genetic causes of familial POI, especially in consanguineous pedigrees [2]. In this study, we used WES to identify a novel mutation for POI in a consanguineous family. Our study is the first to report that mutations in *FIGLA* cause POI in a recessive manner. Our findings will aid researchers and clinicians to understand genetic causes for POI and help patients by genetic counseling.

## Methods

### Patients

Two sisters with POI were recruited from The First Affiliated Hospital of Anhui Medical University. Their parents were consanguineous marriage. POI was diagnosed if the patients had amenorrhea for at least 4 months under the age of 40 and two consecutive follicle stimulating hormone (FSH) measurements > 25 IU/L taken 4 weeks apart [7]. None of the patients showed any of the following: karyotypic abnormality, autoimmune disorder, history of radiotherapy and chemotherapy, or pelvic surgery. Before the patients came to our hospital, they had been on hormone replacement therapy. This study was approved by the Ethics Committee of Anhui Medical University. Written informed consent was obtained from all participants, and peripheral blood (5 mL) was subsequently collected from each participant.

### WES and sanger sequencing validation

WES was performed as previously described [8]. WES was carried out on the proband. The sequencing raw reads were 32,530,181, and the raw depth (×) was 161.44. Sanger sequencing was performed using gene-specific primers. Firstly, the PCR product containing the *FIGLA*’s mutation site was amplified using the specific primers as follow, 5’-GCGAGAAAGAGGGCAACG-3′ for forward primer, and 5’-TGGTGGTAGAGCAGGGAAGG-3′ for reverse primer.

### Bioinformatic analysis of the sequence variant

Three online prediction tools were used in this study to predict the pathogenicity of the variant. For Mutation Taster (http://www.mutationtaster.org/), the probability value is the probability of the prediction, i.e., a value close to 1 indicates a high ‘security’ of the prediction. For SNPs&GO (http://snps.biofold.org/snps-and-go/), if the value was more than 0.5, the mutation is predicted as Disease. For FATHMM-MKL (http://fathmm.biocompute.org.uk/fathmmMKL.htm), values above 0.5 are predicted to be damaging, while those below 0.5 are predicted to be neutral or benign. The allele frequency was evaluated by searching the human exome-seq or whole genome-seq databases such as Exome Aggregation Consortium (ExAC, http://exac.broadinstitute.org/) and genome Aggregation Database (gnomAD, http://gnomad.broadinstitute.org/). ExAC contained 60,706 unrelated individuals’ exome sequencing data, and gnomAD is a big database containing 123,136 exome sequences and 15,496 whole-genome sequences.

## Results

### Clinical features of the family

**Fig. 1:**
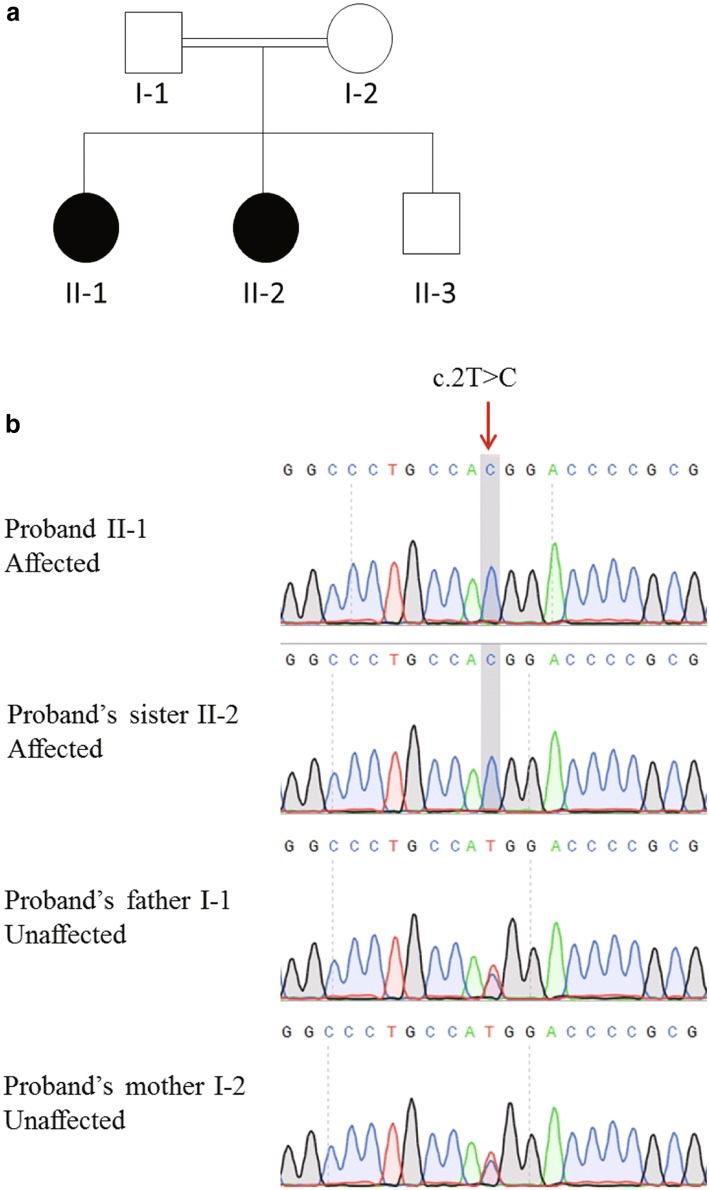
Pedigree and genetic analysis of the patients in family 1. (**a**) Two POI patients in a Chinese consanguineous pedigree. The black circle indicates the affected family members. (**b**) Sanger sequencing validation of the mutation in family members. Red arrow indicates the mutation site

Both the proband (II-1, Fig. 1a) and her younger (II-2) were diagnosed as POI with primary amenorrhea. The proband’s father and mother were in a consanguineous marriage. The proband’s paternal grandfather and maternal grandmother were siblings. The proband was 31 years old when diagnosed with POI in 2011. Her height was 172 cm and weight was 75 kg. Hormone levels of the proband were as follows: FSH, 36.81 IU/L; luteinizing hormone (LH), 10.2 IU/L; testosterone (T), 1.47 nmol/L; estradiol (E2), 122 pmol/L; prolactin (PRL), 8.8 ng/mL; and anti-Müllerian hormone (AMH), 0.05 ng/mL. The proband’s ovaries were not detected by transvaginal color Doppler ultrasound examination. The hormone levels of the proband’s sister were as follows: FSH, 54.49 IU/L; LH, 16.29 IU/L; T, 0.43 nmol/L; E2, 14 pmol/L; and PRL, 10.46 ng/mL. The proband’s mother (I-2) underwent menopause at the age of 53. The proband’s brother (II-3) was fertile and had twin daughters.

### Whole-exome sequencing analysis

To identify the genetic cause for POI in these two sisters, we first set an analysis pipeline. Each patient underwent WES, with sequence reads aligned and variants called, as previously described [8]. Pedigree analysis suggested a recessive mode of inheritance. Likely causative mutations were identified after filtering polymorphisms with allele frequencies > 1% in Exome Aggregation Consortium (ExAC), Genome Aggregation Database (gnomAD), 1000 Genomes and dbSNP databases. A list of genes harboring homozygous variants was further filtered by the functional impact of the mutation, e.g., conservation and functional prediction. Gene relevance for ovarian function was also assessed using gene expression data (Human Protein Atlas, http://www.proteinatlas.org/) and mouse model phenotype data (Mouse Genome Informatics, http://www.informatics.jax.org/).

According to pipeline and several filtering steps after WES, only the *FIGLA* (folliculogenesis specific bHLH transcription factor) gene and its homozygous mutation, NM_001004311:exon1:c.2 T > C, was identified in the proband. This homozygous mutation was also harbored by the proband’s sister and was segregated within the pedigree (Fig. 1b). *FIGLA* has been shown to be specifically expressed in human ovary [9, 10] and was reported to be associated with POI by haploinsufficiency effect [11]. However, our finding did not support the haploinsufficiency effect of *FIGLA* causing POI.

### Bioinformatics analysis of the mutation

**Fig. 2:**
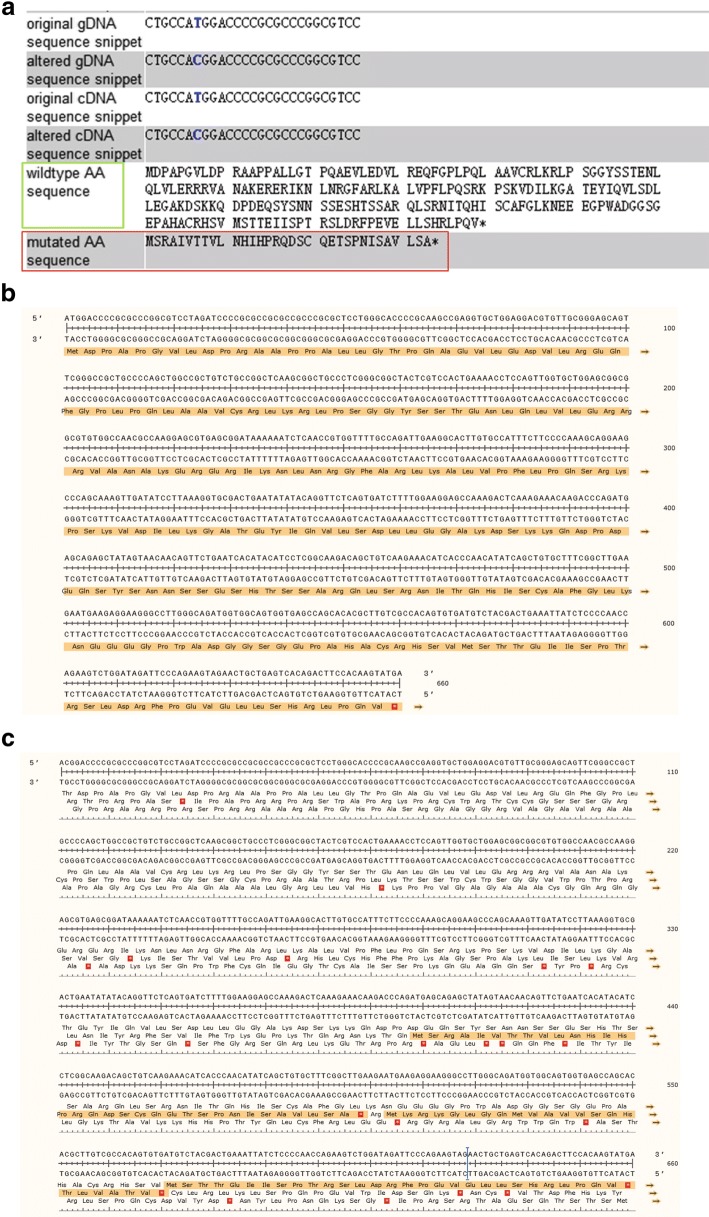
(**a**) In silico analysis of the alternative start site and potential resulting protein. The MutationTaster online prediction tool (http://www.mutationtaster.org/) was used in the current analysis. The predicted results for c.2 T > C showed that the start ATG shifted, the Kozak consensus sequence changed, and the entire protein amino acid sequence frameshifted and was changed. The green box indicates the wild-type amino acid sequence, while the red box indicates the mutated amino acid sequence. (**b**) and (**c**) The wild-type (**b**) and mutated (**c**) sequences of FIGLA. The light grown indicate the putative translated protein sequences

In silico analysis by four online prediction tools suggested that the c.2 T > C mutation was a pathogenic mutation (Table 1). The c.2 T > C mutation lost the start codon and therefore the open reading frame of *FIGLA* was altered (Fig. 2a, b and c). The c.2 T > C mutation was absent in the ExAC, gnomAD, 1000G and dbSNP databases, indicating the extremely rarity of this mutation.

**Table 1:** In silico analysis of the *FIGLA* mutation

Gene	Mutation	Amino acid change	Consanguinity	Zygosity	Mutation Taster^a^	SNPs&GO^b^	FATHMM-MKL^c^	ExAC (total)^d^	ExAC (East Asian)^e^	gnomAD (total)^f^
*FIGLA*	c.2 T > C	Start ATG shift	Yes	Homozygous	Disease causing (1)	Neutral (0.157)	Damaging (0.8119)	0	0	0

^a^Mutation Taster (http://www.mutationtaster.org/). The probability value is the probability of the prediction, i.e., a value close to 1 indicates a high ‘security’ of the prediction

^b^SNPs&GO (http://snps.biofold.org/snps-and-go/). Disease probability (if > 0.5 mutation is predicted Disease)

^c^FATHMM-MKL (http://fathmm.biocompute.org.uk/fathmmMKL.htm). Values above 0.5 are predicted to be deleterious, while those below 0.5 are predicted to be neutral or benign

^d^Frequency of variations in total of ExAC database.

^e^Frequency of variations in East Asian population of ExAC database

^f^Frequency of variation in total of gnomAD (genome Aggregation Database, a big database containing 123,136 exome sequences and 15,496 whole-genome sequences)

## Discussion

Here we report that a novel homozygous mutation in *FIGLA* is associated with POI in one consanguineous pedigree.

*FIGLA* encodes a basic helix-loop-helix transcription factor that regulates primordial follicle formation [12, 13]. FIGLA is specifically expressed in human and mouse ovaries [9, 10, 14]. *Figla* knockout mice cannot form primordial follicles, leading to oocyte loss and female sterility [13]. FIGLA transcriptionally activates oocyte-related genes, such as *ZP1*, *ZP2* and *ZP3* [10, 13], and represses sperm-associated genes during postnatal oogenesis [15].

A previous study found that *FIGLA* haploinsufficiency may be associated with premature ovarian failure [11]. The study found a heterozygous p.G6 fs*66 mutation in a patient with secondary amenorrhea at 36 years of age, and a heterozygous p.140delN mutation in a patient with secondary amenorrhea at 27 years of age [11]. The p.G6 fs*66 mutation creates *FIGLA* haploinsufficiency, because the truncated protein only shares five amino acids with the wild-type protein [11]. The p.140delN mutation disrupts FIGLA binding to TCF3 [11], however no dominant negative effect of p.140delN was observed in that study. So that study did not prove the cause and effect of the variants. While our findings indicate a novel underlying mechanism based on two facts: one is that, the patient’s mother, who is heterozygous for the mutation, was neither a POI patient nor had the phenotype of premature menopause; another is that, the c.2 T > C mutation abolishes the start codon, leading to an alternative open reading frame. The two facts above indicated that loss of function in one *FIGLA* allele can not cause POI. Therefore, our consanguineous pedigree analysis suggests that recessive inheritance of the *FIGLA* mutation is implicated in POI pathogenesis. However, our study did not oppose the previous finding, as the patient with homozygous mutation in our study was primary amenorrhea while the patient with heterozygous mutation in the previous study was secondary amenorrhea. Based on the current results and those of previous studies, we hypothesized that *FIGLA* haploinsufficiency may cause a milder POI than the *FIGLA* homozygous allele mutations.

## Conclusions

Taken together, our study demonstrates biallelic *FIGLA* mutation causing POI with primary amenorrhea. This study will aid researchers and clinicians in genetic counseling of POI and provides new insight into understanding the mode of genetic inheritance of certain genes and POI.
